# Emergence of *Klebsiella pneumoniae* clinical isolates producing KPC-2 carbapenemase in Cuba

**DOI:** 10.1002/nmi2.54

**Published:** 2014-06-26

**Authors:** D Quiñones, M Hart, F Espinosa, S Garcia, Y Carmona, S Ghosh, N Urushibara, M Kawaguchiya, N Kobayashi

**Affiliations:** 1 Servicio de Bacteriología-Micología, Instituto de Medicina Tropical Pedro KouríLa Habana, Cuba; 2 Hospital ‘Hermanos Ameijeiras’La Habana, Cuba; 3 Hospital ‘V. I. Lenin’Holguín, Cuba; 4 Department of Hygiene, Sapporo Medical University School of MedicineSapporo, Japan; 5 Department of Biomedical Sciences, Ross University School of Veterinary MedicineSt. Kitts, West Indies

**Keywords:** Clinical isolates, Cuba, *Klebsiella pneumoniae* producing carbapenemase, multilocus sequence typing

## Abstract

The emergence of *Klebsiella pneumoniae* producing carbapenemase (KPC) has now become a global concern. As a part of a nationwide multicentre surveillance study in Cuba, three *K. pneumoniae* clinical isolates resistant to carbapenems were detected for a 1-month period (September to October 2011). PCR and sequence analysis revealed that the three strains harboured *bla*_KPC-2_. They showed resistance or intermediate susceptibility to expanded-spectrum cephalosporins, other β-lactams, a β-lactam/β-lactamase inhibitor combination, and gentamicin. Two strains were susceptible only to colistin, whereas the other strain showing colistin resistance was susceptible to fluoroquinolones. These *bla*_KPC__-2_-positive *K. pneumoniae* strains were classified into ST1271 (CC29), a novel clone harbouring *bla*_KPC__-2_, and were revealed to be genetically identical by PCR-based DNA fingerprinting. The three patients infected with the KPC-producing *K. pneumoniae* had common risk factors, and had no overseas travel experience outside Cuba, suggesting local acquisition of the resistant pathogen. This is the first report of a KPC-producing *K. pneumoniae* in Cuba. Although detection of KPC in Enterobacteriaceae is still rare in Cuba, our finding indicated that KPC-producing bacteria are a global concern and highlighted the need to identify these microorganisms in clinical laboratories.

## Introduction

The *Klebsiella pneumoniae* carbapenemase (KPC) was first described in 1996 in North Carolina, USA. Thereafter, expansion of KPC in clinical isolates has been reported from different continents associated with the global spread of the clonal lineages of *K. pneumoniae*, such as ST248/ST258 1. The ST258 *K. pneumoniae* has been described as an international KPC-producing clone 2, and its global spread including Latin American countries has also been reported 3. Because the KPC gene is carried by plasmids, potentially rapid transmission of carbapenem resistance has been recognized as a major threat to the antimicrobial treatment of infections with gram-negative microorganisms 2. The Pan American Health Organization issued an epidemiological alert with the increase of carbapenemase in Enterobacteriaceae from many Latin American countries in 2010 4. Since then, Cuba has initiated a surveillance network for *K. pneumoniae* clinical isolates from reference hospitals to analyse their antimicrobial susceptibility and genetic mechanisms of drug resistance with special attention to carbapenem resistance, in the ‘Pedro Kourí’ Institute of Tropical Medicine 5. During this national surveillance, three isolates of KPC-producing *K. pneumoniae* were detected for the first time in Cuba.

## Methods

As a part of a nationwide multicentre surveillance study in Cuba, three *K. pneumoniae* clinical isolates resistant to carbapenems were detected in the ‘Pedro Kourí’ Institute of Tropical Medicine for a 1-month period (September to October 2011). The antimicrobial susceptibility to a wide range of antibiotics was determined using E-test (BioMérieux, Marcy l'Etoile, France) according to the manufacturer's recommendation. MICs were interpreted into susceptible or resistant according to CLSI guidelines, 2012 6, except for colistin, which was judged by EUCAST criteria (susceptible ≤2 g/mL, resistant ≥4 g/mL), (http://www.eucast.org/clinicalbreakpoints/). A double-disc synergy test was performed to detect extended spectrum β-lactamases (ESBLs) 6 and 3-aminophenylboronic acid test was used to screen for production of carbapenemases 7. The presence of genes encoding carbapenemase was determined by PCR using protocols and conditions as described previously 8. Nucleotide sequence of *bla*_KPC_ was determined by direct sequencing with PCR products by using the BigDye Terminator version 3.1 cycle sequencing kit (Applied Biosystems, Foster City, CA, USA). Sequence type (ST) of *K. pneumoniae* based on the scheme of multilocus sequence typing (MLST) was determined according to the methods available at the website (www.pasteur.fr/recherche/genopole/PF8/mlst/Kpneumoniae.html). KPC-2 gene sequence of *K. pneumoniae* strain 354 was deposited to GenBank database under accession no. KJ151293. To analyse genetic diversity of *K. pneumoniae* isolates, genomic DNA fingerprinting was performed by randomly amplified polymorphic DNA analysis with the use of a single primer (M13, ERIC-2, AP-1, AP-4 and AP-7), and repetitive extragenic palindromic sequence-based PCR (REP-PCR) with a pair of primers (REP1R-I, REP2-I), as described previously 9–13.

## Results and Discussion

**Figure 1:**
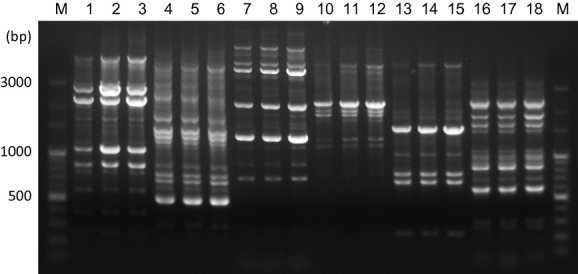
DNA fingerprints obtained by randomly amplified polymorphic DNA analysis with primer M13 (lanes 1–3), ERIC2 (lanes 4–6), AP-1 (lanes 7–9), AP-4 (lanes 10–12), and AP-7 (lanes 13–15), and repetitive extragenic palindromic sequence-based PCR (lanes 16–18). Lanes 1, 4, 7, 10, 13, 16, strain 328; lanes 2, 5, 8, 11, 14, 17, strain 354; lanes 3, 6, 9, 12, 15, 18, strain 355. Lane ‘M’ represents DNA size marker and molecular size (bp) is shown on the left.

Three clinical isolates of *K. pneumoniae* (strains 328, 354, 355) recovered from two provinces of Cuba (Holguin and Havana city) were confirmed to be resistant to imipenem and meropenem. These strains showed resistance or intermediate susceptibility to expanded-spectrum cephalosporins, *β*-lactam, β-lactam/β-lactamase inhibitor combination (piperacillin/tazobactam), and gentamicin. Two of these strains (354, 355) were susceptible only to colistin, whereas the other strain (328) showing resistance to colistin was susceptible to fluoroquinolones (Table1). The synergy test to detect ESBLs and carbapenemase was positive, suggesting the production of an ESBL and KPC enzyme. The KPC gene was detected in the three strains by PCR, and their sequences were revealed to be identical to *bla*_KPC-2_. The three *bla*_KPC-2_-positive *K. pneumoniae* strains were classified into ST1271 (CC29), a single locus variant of ST29. The ST1271 was identified as a novel clone harbouring *bla*_KPC-2_ in the present study, because the presence of *bla*_KPC-2_ had not been reported in ST1271 as well as ST29 clones. By randomly amplified polymorphic DNA analysis with five different primers and REP PCR, three strains showed the same banding patterns (Fig.1), indicating that these strains are genetically identical and have the same origin. The two colistin-susceptible strains were derived from the same hospital in Havana, exhibited similar resistance patterns, and are suggested to be the same strain that had transmitted via nosocomial infection.

**Table 1:** Antimicrobial susceptibility of *Klebsiella pneumoniae* clinical isolates producing KPC-2 carbapenemase

Antibiotics	MIC Interpretation^a^		
	Strain 328	Strain 354	Strain 355
Piperacillin/tazobactam	R	R	R
Cefotaxime	R	R	R
Ceftazidime	R	R	R
Cefoxitin	S	R	R
Cefepime	R	R	R
Aztreonam	R	R	R
Meropenem	R	R	R
Imipenem	R	R	I
Amikacin	R	R	I
Gentamicin	R	R	R
Colistin	R	S	S
Ciprofloxacin	S	R	R
Levofloxacin	S	R	R
Nalidixic acid	S	R	R
Trimethoprim-sulfamethoxazole	R	R	R

R, Resistant; S, susceptible; I, Intermediate.

a According to CLSI standards (2012).

The three strains with KPC genes were derived from two separate provinces, i.e. Holguin (eastern Cuba) and Havana city (western Cuba). Patients infected with the KPC-producing *K. pneumoniae* had no experience of travel outside Cuba, suggesting that local acquisition and a silent dissemination of the KPC-positive *K. pneumoniae* ST1271 clone in this country. Because intercontinental travel has been directly linked with the spread of KPCs through patients colonized or infected with KPC-producing *K. pneumoniae* 14,15, international tourism might have played an important role in its emergence in Cuba. ST29 *K. pneumoniae* strains have been isolated from Europe, e.g. Spain and Greece, and also from Brazil 3. In our previous report on the antimicrobial resistance of *K. pneumoniae* in Cuba through the national surveillance programme (2009–10), 54 isolates (23.6%) were positive for ESBL, and were classified into 27 STs, showing high clonal diversity 5. However, none of the ST identified in the ESBL-positive isolates belonged to CC29. Therefore, in Cuba, the ST1271 *K. pneumoniae* strains are genetically distinct from ESBL-positive strains. Although the origin of the ST1271 strains has yet to be determined, it is possible that ST1271 might have been brought from another country recently, associated with acquisition of the KPC gene. Further molecular epidemiological studies on *K. pneumoniae* isolates, including those from healthy individuals, may provide more information on distribution and transmission of the ST1271 clone.

It was noted in the present study that an ST1271 *K. pneumoniae* strain showed resistance to colistin. Generally, the colistin resistance rate of *K. pneumoniae* has been reported to be low 16. During the period of antimicrobial resistance surveillance (2010–12) of *Klebsiella* spp. in Cuba, we notified 15% colistin resistance, which would be of great concern in clinical settings 17. Our present finding indicates that the colistin resistance has emerged in multidrug-resistant *K. pneumoniae*. This antibiotic is recognized as a key therapeutic option for carbapenem-resistant bacteria, and is particularly important in countries with limited resources, such as Cuba where tigecycline is not available.

Table2 shows the clinical information of three patients infected with *K. pneumoniae* producing KPC-2 carbapenemase. These patients had common risk factors, such as prolonged hospitalization, intravenous catheter, previous antimicrobial therapy, and underlying disease. These findings are consistent with previous studies that notified risk factors for acquiring infections with KPC-producing *K. pneumoniae* 18. In the present study, all three *K. pneumoniae* producing KPC were isolated from blood specimens and two of the patients died with septic shock. The susceptibility profile of these isolates indicated considerably limited therapeutic options, i.e. colistin in two patients or fluoroquinolone in one patient. The optimal treatment for infections caused by KPC-producing *K. pneumoniae* has been difficult to describe, resulting in mortality rates of at least 50% 19. Therefore, evaluation of effective antibiotic options and rigorous infection control measures may be necessary to reduce carbapenemase-producing microorganisms.

**Table 2:** Clinical information of patients infected with *Klebsiella pneumoniae* producing KPC-2 carbapenemase

Cases	Age	Sex	Province	Hospital	Department	Isolation date	Risk factors	Disease	Specimen	Final therapy	Died
Case-1 (strain 328)	42	Male	Holguín (Eastern Cuba)	1	Intensive care unit	September 2011	Previous antimicrobial therapy, urinary catheter, prolonged hospitalization, intravenous catheter disease (Haemorrhage itraparenkimatosa)	Septicaemia	Blood	Ciprofloxacin	No
Case-2 (strain 354)	49	Female	Havana city (Western Cuba)	2	Marrow transplant unit	October 2011	Previous antimicrobial therapy, chemotherapy gynaecological surgery prolonged hospitalization, intravenous catheter Underlying disease (Acute myeloid leukaemia)	Cellulitis in surgery site Septic shock	Blood	Cefepime, amikacin, vancomycin and amphotericin B	Yes
Case-3 (strain 355)	57	Female	Havana city (Western Cuba)	2	Intensive care unit	October 2011	Previous antimicrobial therapy, surgery for valve replacement, mechanical ventilation urinary catheter, prolonged hospitalization, intravenous catheter, Underlying disease (Mitral stenosis)	Pneumonia Paralytic ileus Cardiogenic shock Septic shock	Blood	Colistin plus amphotericin B	Yes

KPC, *Klebsiella pneumoniae* producing carbapenemase.

Detection of the novel KPC-producing *K. pneumoniae* clone (ST1271) in the present study demonstrated the importance of monitoring hospitalized patients for the further emergence of carbapenem resistance in *K. pneumoniae* as well as in other gram-negative pathogens. Although carbapenemase-producing Enterobacteriaceae are still rare in Cuba, our finding confirmed that KPC-producing isolates are a global concern, highlighting the need to identify these microorganisms in clinical laboratories.
